# Identification and characterization of mitochondrial autophagy-related genes in osteosarcoma and predicting clinical prognosis

**DOI:** 10.1038/s41598-025-95173-w

**Published:** 2025-03-24

**Authors:** Hongliang Zhang, Jingyu Zhang, Kai Zhu, Shuang Li, Jinwei Liu, Boya Guan, Hong Zhang, Changbao Chen, Yancheng Liu

**Affiliations:** 1 Department of Bone and Soft Tissue Tumor, Tianjin Hospital, Tianjin University, Tianjin, 300211 China; 2 Department of Pharmacy, Tianjin Hospital, Tianjin University, Tianjin, 300211 China; 3 Department of Spinal Surgery, Tianjin Hospital, Tianjin University, Tianjin, 300211 China

**Keywords:** Osteosarcoma, Mitochondrial autophagy, Prognostic genes, Risk model, Biomarkers, Oncology

## Abstract

Osteosarcoma (OS), the most prevalent primary malignant bone tumor, is characterized by a poor prognosis and high metastatic potential. Mitochondrial autophagy has been implicated in cancer suppression. This study aimed to identify prognostic genes associated with mitochondrial autophagy in OS. Public datasets, including TARGET-OS, GSE99671, and GSE21257, were retrieved for analysis. Differentially expressed genes (DEGs1) between OS and normal samples were identified from GSE99671. Single-sample Gene Set Enrichment Analysis (ssGSEA) was applied to quantify the enrichment scores of 29 mitochondrial autophagy-related genes (MARGs) in OS samples from TARGET-OS, categorizing them into high- and low-score groups to extract DEGs2. The intersection of DEGs1 and DEGs2 yielded mitochondrial autophagy-associated differentially expressed genes (MDGs). Prognostic genes were subsequently screened through a multi-step regression analysis, and a risk score was computed. TARGET-OS samples were stratified into high- and low-risk groups based on the optimal cutoff value of the risk score. GSEA was conducted between the two risk groups. Additionally, associations between prognostic genes and the immune microenvironment were explored. A total of 31 MDGs were identified from the overlap of 3,207 DEGs1 and 622 DEGs2. Five prognostic genes—KLK2, NRXN1, HES5, OR2W3, and HS3ST4—were further selected. Kaplan-Meier survival analysis indicated significantly reduced survival in the high-risk group. GSEA revealed enrichment in ABC transporter activity and glycolysis/gluconeogenesis pathways. Immunoanalysis demonstrated significant differences in 11 immune cell populations and three immune functions between risk groups, notably myeloid-derived suppressor cells (MDSCs) and Type 1 T helper cells. HS3ST4 exhibited the strongest positive correlation with macrophages, whereas NRXN1 showed the most pronounced negative correlation with memory B cells. Expressions of HAVCR2 and PDCD1LG2 were elevated in the low-risk group. Functional analysis indicated significant differences in dysfunction patterns between risk groups. This study identified five mitochondrial autophagy-related prognostic genes and constructed a risk model, offering novel insights into OS diagnosis and therapeutic strategies.

## Introduction

Osteosarcoma (OS), the most prevalent primary malignant bone tumor, is believed to originate from malignant mesenchymal stem cells^1^. Its annual incidence is approximately 2–3 cases per million in the general population^2^. OS predominantly affects rapidly growing adolescents and individuals over 60 years old with Paget’s disease, with the metaphysis of long bones being the most frequent site of occurrence^3^. The current standard treatment comprises neoadjuvant chemotherapy (doxorubicin, cisplatin, and high-dose methotrexate), radical surgical resection, and adjuvant chemotherapy^4^. Additionally, immunotherapy and radiotherapy have emerged as important therapeutic modalities for OS^5^. Despite advancements in perioperative multimodal therapy, the 5-year survival rate for patients with localized appendicular tumors remains at 60–70%, and further improvements in chemotherapy efficacy have stagnated over recent decades^6^. At diagnosis, 10–15% of patients with OS present with metastases, predominantly in the lungs. Prognosis remains dismal for those with metastatic or recurrent disease, with the 5-year survival rate plummeting to below 20%^7^. Given the urgent need to elucidate OS pathogenesis and identify novel therapeutic targets, the discovery and validation of prognostic biomarkers have become imperative in clinical practice.

Mitochondrial autophagy, a highly conserved cellular process, plays a pivotal role in preserving cellular homeostasis by selectively degrading dysfunctional mitochondria. In response to various stressors, including reactive oxygen species (ROS), nutrient deprivation, and cellular senescence, mitochondrial autophagy facilitates the removal of impaired mitochondria through autophagosome formation, followed by lysosomal degradation^4^. This process is essential for maintaining mitochondrial network integrity and cell viability, while its dysregulation is implicated in numerous pathological conditions, including malignancies, cardiovascular disorders, metabolic syndromes, and neurodegenerative diseases^8^. Recent studies have highlighted mitochondrial autophagy as a critical factor in cancer biology. Key regulatory genes, such as PINK1, Parkin, FUNDC1, PHB2, and BNIP3, have emerged as potential therapeutic targets for overcoming drug resistance in cancer treatment^9^. Notably, mitochondrial autophagy has been linked to hepatocellular carcinoma suppression^10^. By sustaining mitochondrial homeostasis, it promotes tumor cell proliferation, whereas its dysfunction disrupts metabolic balance and induces excessive ROS accumulation, ultimately triggering tumor cell apoptosis. Furthermore, silencing PINK1, Parkin, FUNDC1, and BNIP3 has been shown to enhance tumor cell migration and invasion across various malignancies^11^. These findings suggest that mitochondrial autophagy may be intricately involved in the pathogenesis and progression of OS.

Bioinformatics methods have recently been employed to predict disease target genes and analyze their potential molecular mechanisms, thereby providing more feasible ideas and protocols for subsequent trials. The primary goals of these studies are to elucidate a deeper understanding of disease pathogenesis and to investigate novel target drugs, particularly in light of advancements in gene chip and high-throughput sequencing technologies. Researchers in the field of cancer are particularly interested in utilizing MARGs as diagnostic or prognostic molecular biomarkers^9–11^. However, the prognostic roles of MARGs and their biological functions in osteosarcoma remain largely unknown. Furthermore, a reliable MARGs signature for predicting overall survival has not yet been identified.

In this study, OS-related transcriptomic and clinical data were retrieved from public databases. Multiple regression analyses were performed to identify mitochondrial autophagy-associated prognostic genes and develop a risk model for OS survival prediction. Additionally, the relationship between these prognostic genes and the tumor immune microenvironment, as well as their potential relevance in immunotherapy, was explored. Finally, gene expression was validated *via* reverse transcription-quantitative polymerase chain reaction (RT-qPCR), providing a theoretical foundation for OS diagnosis and therapeutic strategies.

## Methods

### Patients cohorts

Survival data for TARGET-OS samples were obtained from the University of California, Santa Cruz (UCSC) Xena platform (https://xena.ucsc.edu/) and utilized for risk model construction. Patients with OS without survival data were excluded, resulting in a final cohort of 85 patients with OS having complete survival and gene expression data for analysis. Additional datasets, GSE99671 and GSE21257, were retrieved from the Gene Expression Omnibus (GEO) database (https://www.ncbi.nlm.nih.gov/geo/). GSE99671, comprising 18 OS and 18 normal bone samples, was used for differential expression analysis, while GSE21257, containing survival and gene expression data for 53 patients with OS, served as an independent validation set for the risk model. Furthermore, 29 MARGs were extracted from the Reactome database (https://reactome.org/), encompassing pathways such as mitochondrial autophagy (R-HSA-5205647), PINK1-PRKN-mediated mitochondrial autophagy (R-HSA-5205685), and receptor-mediated mitochondrial autophagy (R-HSA-8934903).

Five pairs of matched OS and adjacent normal tissue samples were collected from Tianjin University of Tianjin Hospital during surgical procedures for RT-qPCR validation. Informed consent was obtained from all participants, and the study was approved by the Tianjin University of Tianjin Hospital Ethics Committee. All experimental procedures adhered to relevant ethical guidelines and regulations.

### Differential analysis and enrichment analysis

In GSE99671, differentially expressed genes (DEGs1) between OS and normal samples were identified on the count data using DESeq2^12^, applying a significance threshold of *P* < 0.05 and |log_2_fold change (FC)| > 0.5. A volcano plot and heatmap of DEGs were generated using the ggplot2^13^ and ComplexHeatmap^14^ packages, respectively. Subsequently, single-sample gene set enrichment analysis (ssGSEA) was performed using GSVA^15^ to quantify the expression scores of 29 MARGs in TARGET-OS samples. Based on the optimal threshold, OS samples were stratified into high- and low-score groups, and DESeq2 was employed to identify DEGs2 between these groups, using the same statistical criteria (*P* < 0.05 and |log_2_FC| > 0.5). The intersection of DEGs1 and DEGs2 yielded mitochondrial autophagy-related differentially expressed genes (MDGs). To explore the biological functions and signaling pathways associated with MDGs, enrichment analysis was conducted using clusterProfiler^16^, focusing on Gene Ontology (GO) terms and Kyoto Encyclopedia of Genes and Genomes (KEGG) pathways^17–19^ (*P*.adjust < 0.05).

### Construction and validation of the risk model

To investigate the effect of candidate genes on the survival of OS patients, univariate Cox regression analysis was conducted on MDGs using the survival^20^ package, with survival-associated genes identified based on hazard ratios (HR) ≠ 1 and *P* < 0.05. Genes with HR > 1 were classified as high-risk, whereas those with HR < 1 were considered low-risk. Subsequently, least absolute shrinkage and selection operator (LASSO) regression was performed using glmnet^21^ to refine prognostic gene selection, with 10-fold cross-validation determining the optimal lambda value. Genes with nonzero regression coefficients were retained as prognostic markers, forming the basis of the risk model. Risk scores were calculated, and patients with OS in TARGET-OS and GSE21257 were stratified into high- and low-risk groups based on the optimal risk score cutoff. Kaplan-Meier (K-M) survival analysis was then employed to compare survival differences between risk groups. Receiver operating characteristic (ROC) curves for 1-, 3-, and 5-year survival were plotted separately for TARGET-OS and GSE21257 using the survivalROC package (v1.0.3.1, v1.0.3.1 https://CRAN.R-project.org/package=survivalROC) to evaluate the model’s predictive accuracy.

### Construction and evaluation of the nomogram

To facilitate an intuitive prognostic assessment, a nomogram was developed. The risk model was integrated with clinical variables, followed by univariate Cox regression analysis (*P* < 0.05) of four factors—risk score, age, gender, and tumor stage (metastatic vs. non-metastatic)—to identify significant prognostic indicators. Factors meeting the *P* < 0.05 threshold underwent the Proportional Hazards (PH) assumption test and multivariate Cox regression analysis to determine independent prognostic factors. A nomogram incorporating these factors was then constructed to predict 1-, 3-, and 5-year survival probabilities in patients with OS, with calibration curves assessing its predictive performance.

### GSEA of distinct risk groups

DESeq2 was applied to identify differences in enrichment pathways between high and low risk groups in TARGET-OS, followed by log_2_FC and ranking. The gene set c2.cp.v2023.2.Hs.symbols.gmt was retrieved from The Molecular Signatures Database (MSigDB) (https://www.gsea-msigdb.org/gsea/msigdb/), and GSEA was conducted, with *P* < 0.05 considered statistically significant. GSEA results were visualized using the enrichplot^22^ package.

### Immunoinfiltration analysis

To examine the association between prognostic genes and the OS immune microenvironment, immune infiltration analysis was performed in TARGET-OS. The ssGSEA algorithm was employed to quantify the infiltration levels of 28 immune cell types and immune function scores across OS samples, comparing differences between risk groups. Correlation analyses were then conducted to evaluate relationships between differentially infiltrated immune cells, immune functions, and prognostic gene expression.

### Immune checkpoint and tumor immune dysfunction and exclusion (TIDE) analysis

20 immune checkpoint genes (ADORA2A, ARHGEF5, BTLA, CD160, CD244, CD27, CD274, CD276, CD47, CD80, CEACAM1, CTLA4, GEM, HAVCR2, ICOS, IDO1, LAG3, PDCD1, TNFSF4, VTCN1) were identified from the literature^23^, and their differential expression between high- and low-risk groups was analyzed using the Wilcoxon test. Spearman correlation analysis was then performed to assess the association between differentially expressed immune checkpoints and risk scores. Additionally, tumor immune dysfunction and exclusion (TIDE) scores for patients in TARGET-OS were obtained from the TIDE website (http://tide.dfci.harvard.edu/). Differences in TIDE scores between risk groups were compared, followed by correlation analysis between TIDE scores and risk scores.

### Regulation mechanism and expression verification of prognostic genes

To investigate the regulatory role of prognostic genes in OS, a competitive endogenous RNA (ceRNA) network was constructed. Prognostic gene-associated miRNAs were predicted using The Encyclopedia of RNA Interactomes (ENCORI, http://starbase.sysu.edu.cn/index.php), miRDB (http://www.mirdb.org), and miRWalk (http://mirwalk.umm.uni-heidelberg.de), with overlapping predictions forming the final miRNA set. ENCORI was then utilized to predict lncRNAs, generating miRNA-lncRNA interaction pairs. The ceRNA network was constructed based on prognostic gene interactions. Furthermore, the miRNet database (https://www.mirnet.ca) was used to predict transcription factors (TFs) and miRNAs interacting with prognostic genes. Network visualization was performed using Cytoscape^24^. Additionally, the count data from the GSE99671 dataset was converted to FPKM (Fragments Per Kilobase Million) data, and the differential expression of prognostic genes between OS and normal samples was validated.

### RT-qPCR analysis

Total RNA was extracted from 10 tissue samples using TRIzol reagent (Invitrogen, China) following the manufacturer’s protocol. RNA concentrations were measured with a NanoPhotometer N50. cDNA was synthesized *via* reverse transcription using the SureScript First-Strand cDNA Synthesis Kit (Servicebio, China). RT-qPCR was conducted on a CFX Connect Thermal Cycler (Bio-Rad, USA), and relative mRNA expression levels were calculated using the 2^−ΔΔCT^ method. Primer sequences are listed in Table 1.

**Table 1:** Sequence of primers for prognostic genes.

Gene	Primers sequence (5′−3′)
KLK2	F: TGTGAGCCTCCATCTCCTGT
KLK2	R: CCCTTTCCCCTCCAGATGTTG
NRXN1	F: GAGCTCAGCCAACCCAACC
NRXN1	R: CGGCTACTATCCCAACGACC
HES5	F: GCACCAGGACTACAGCGAAG
HES5	R: GCTGGAAGTGGTACAGCAGC
OR2W3	F: ATCGAAGGCACCGTCTTTGT
OR2W3	R: CTGGGAAGAACTGGCTCCTG
HS3ST4	F: TGAGCGACTCATTGTGGACC
HS3ST4	R: CCGACCTTTGCTCTTGCCTA
GAPDH	F: CGAAGGTGGAGTCAACGGATTT
GAPDH	R: ATGGGTGGAATCATATTGGAAC

### Statistical analysis

All statistical analyses were performed in R (v4.1.0), with *P* < 0.05 considered statistically significant.

## Results

### Identification and functional enrichment of 31 MDGs

**Fig. 1:**
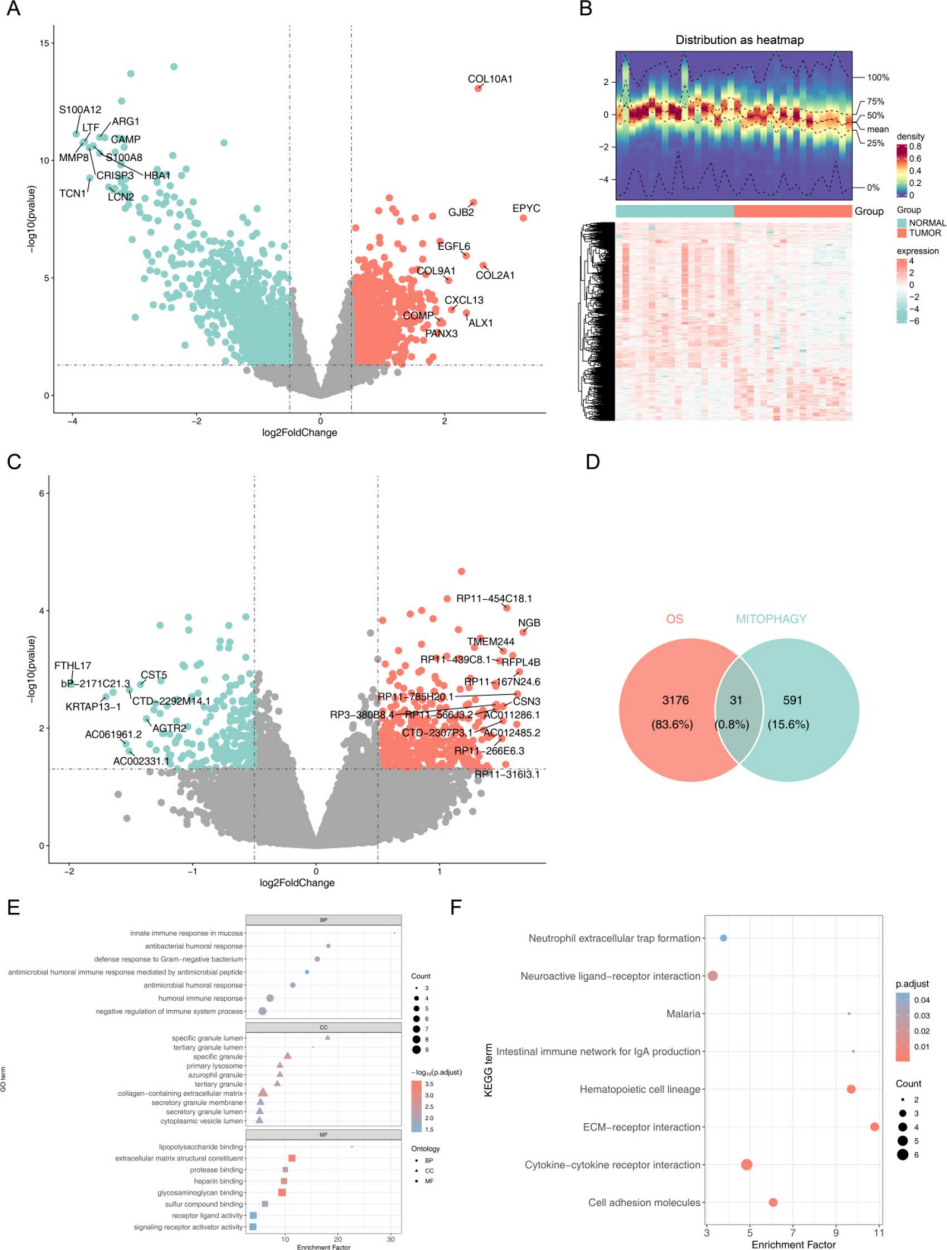
Identification and enrichment analysis of MDGs. (**A**) Volcano plot of DEGs1 in GSE99671, with upregulated genes in orange, downregulated genes in green, and non-significant genes in gray. (**B**) Heatmap of DEGs1, the upper part represented the expression density heatmap of DEGs across samples, displaying the lines for the five percentiles and the mean value, the lower part showed the expression levels of DEGs1 in different samples. The heatmap was generated using the R package “ComplexHeatmap” (version 1.15.1, https://github.com/jokergoo/ComplexHeatmap). (**C**) Volcano plot of DEGs2 between high- and low-MARG score groups in TARGET-OS, with color coding similar to A. (**D**) Venn diagram illustrating the intersection of DEGs1 and DEGs2 to identify MDGs. (**E**) GO enrichment analysis of MDGs, showing significantly enriched terms (adjusted *P* < 0.05). (**F**) KEGG pathway analysis of MDGs, highlighting significantly enriched pathways (adjusted *P* < 0.05). Abbreviations: DEGs, differentially expressed genes; OS, osteosarcoma; MARGs, mitochondrial autophagy-related genes; MDGs, mitochondrial autophagy-related differentially expressed genes; GO, Gene Ontology; KEGG, Kyoto Encyclopedia of Genes and Genomes.

A total of 3,207 DEGs1 were identified between OS and normal samples, comprising 1,751 downregulated and 1,456 upregulated genes (Fig. 1A–B). Additionally, 622 DEGs2 were detected between high- and low-MARG score groups, including 403 upregulated and 219 downregulated genes (Fig. 1C). The intersection of DEGs1 and DEGs2 yielded 31 MDGs (Fig. 1D). GO enrichment analysis revealed that MDGs were primarily associated with negative regulation of immune system processes, cytoplasmic vesicle lumen, and signaling receptor activator activity (Fig. 1E). KEGG pathway analysis further indicated that MDGs were involved in neuroactive ligand-receptor interactions and neutrophil extracellular trap formation (Fig. 1F).

### Identification of five prognostic genes and establishment of risk model

**Fig. 2:**
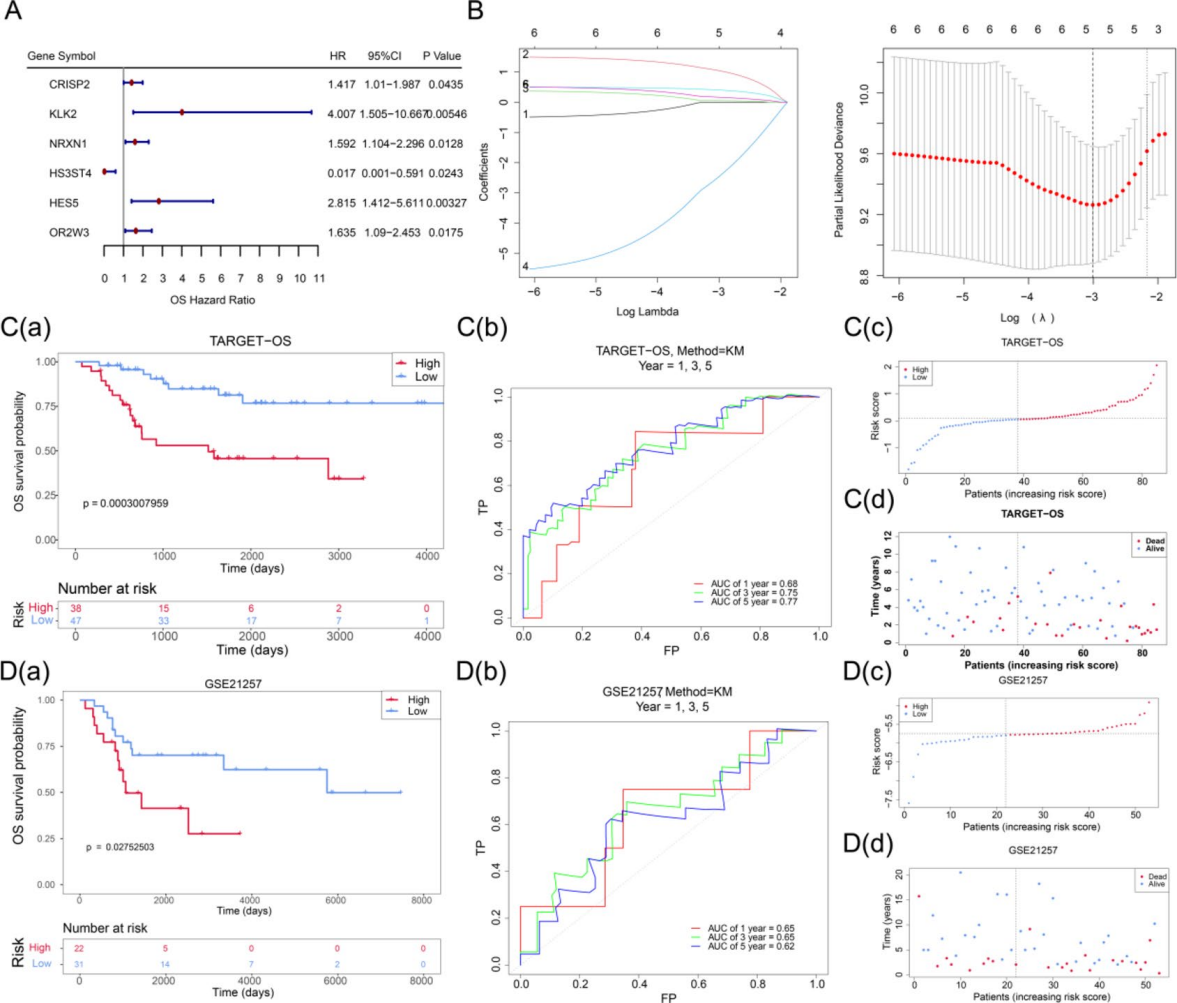
Construction of the Prognostic Gene-Based Risk Model (**A**) Forest plots illustrating the univariate Cox hazard model for prognostic genes significantly associated with overall survival (HR ≠ 1, *P* < 0.05). (**B**) The plot of the LASSO model construction, On the left was the coefficient path plot for the six genes. On the right was the ten-fold cross-validation for adjusting the parameters. The optimal lambda value was chosen at the lowest point of the red curve, with the gray lines representing the SE. The bottom of the vertical line indicated the optimal value. (**C**) TARGET-OS cohort: C(a) Kaplan-Meier survival curves comparing high- and low-risk groups; C(b) ROC curves for predicting 1-, 3-, and 5-year overall survival; C(c) Risk score distribution, with red indicating high risk and blue indicating low risk; C(d) Scatter plot illustrating survival status, where red represents deceased and blue represents surviving patients. (**D**) GSE21257 cohort: D(a) Kaplan-Meier survival curves for high- and low-risk groups; D(b) ROC curves for 1-, 3-, and 5-year overall survival prediction; D(c) Risk score distribution, with high-risk patients in red and low-risk patients in blue; D(d) Survival status plot, where red denotes deceased individuals and blue denotes survivors. Abbreviations: MDGs: mitochondrial autophagy-related differential genes; LASSO: least absolute shrinkage and selection operator; SE: standard error; KM: Kaplan-Meier; ROC: receiver operating characteristic; OS: osteosarcoma.

Univariate Cox regression analysis of candidate genes identified six survival-related genes, among which CRISP2, KLK2, NRXN1, HES5, and OR2W3 were classified as high-risk genes (HR > 1), while HS3ST4 was categorized as a low-risk gene (HR < 1) (Fig. 2A). The optimal lambda value of 0.04936279 was selected *via* LASSO regression, yielding five genes with nonzero regression coefficients: KLK2, NRXN1, HES5, OR2W3, and HS3ST4 (Fig. 2B). A risk model was constructed based on these five genes, with the final risk model was formulated as follows: RiskScore = 1.0475 × KLK2 + 0.0518 × NRXN1-2.4732*HS3ST4 + 0.4032 × HES5 + 0.1656 × OR2W3.

Patients from TARGET-OS (optimal cutoff: 0.09119638) and GSE21257 (optimal cutoff: -5.751265) were stratified into high- and low-risk groups accordingly. Kaplan-Meier survival analysis demonstrated that high-risk patients exhibited significantly shorter survival durations in both datasets (TARGET-OS: *P* = 0.0003, GSE21257: *P* = 0.0275) (Fig. 2Ca, Da). Additionally, the area under the curve (AUC) values for 1-, 3-, and 5-year survival in TARGET-OS (Fig. 2Cb) and GSE21257 (Fig. 2Db) exceeded 0.6, indicating robust predictive performance of the model. Furthermore, a trend of increasing mortality and reduced survival time was observed as risk scores increased in both datasets (Fig. 2Cc-d, Dc-d).

### Establishment of alignment chart with risk score and stage

**Fig. 3:**
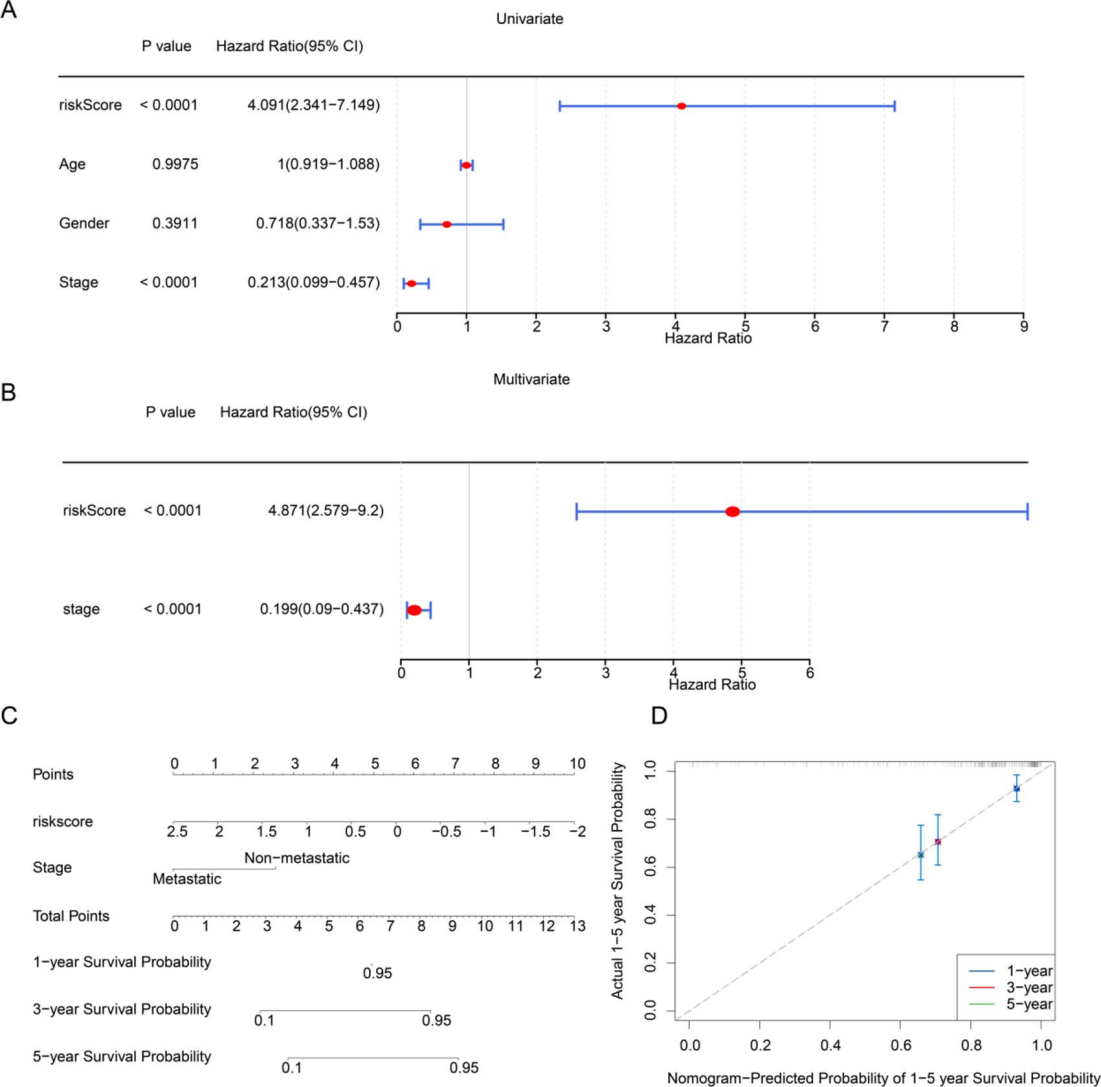
Independent prognostic analyses and nomogram construction. (**A**) Forest plot of clinical indicators in univariate Cox regression analysis. (**B**) Forest plot of clinical indicators in multivariate Cox regression analysis. (**C**) Nomogram incorporating independent prognostic factors. (**D**) The calibration curves of the nomogram.

Univariate Cox regression analysis of pathological indicators and risk score identified risk score (*P* < 0.0001) and tumor stage (*P* < 0.0001) as significant independent prognostic factors for OS survival (Fig. 3A). Both variables satisfied the PH assumption and were further validated through multivariate Cox regression analysis (Fig. 3B). Based on these findings, a nomogram integrating tumor stage and risk score was developed to predict the 1-, 3-, and 5-year survival rates of patient (Fig. 3C). The calibration curve demonstrated a high degree of concordance with the ideal reference line, indicating that the nomogram accurately predicts survival outcomes in patients with OS (Fig. 3D).

### Enrichment pathways and immunological associations between high- and low-risk groups

**Fig. 4:**
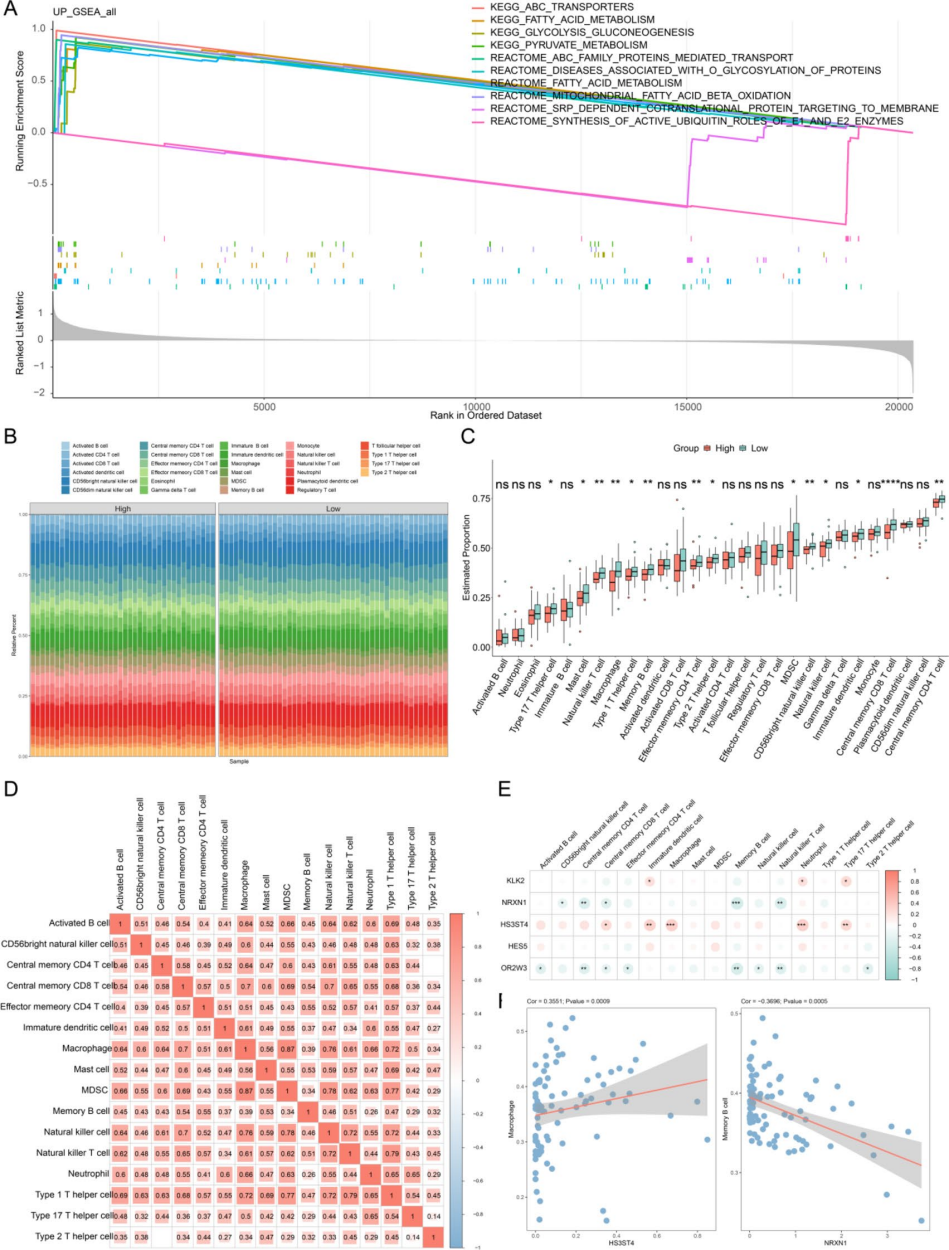
Enrichment pathways and immune cell infiltration across risk groups. (**A**) GSEA highlighting differentially expressed genes between the two risk groups. (**B**) Heatmap illustrating the distribution of immune cell infiltration and functional activity across samples from distinct risk groups. (**C**) The box plots of immune infiltration levels and immune function scores in high and low-risk groups were presented. ns represented no significance; *P < 0.05; **P < 0.01; ****P < 0.0001. (**D**) Heatmap depicting correlations among differentially enriched immune cell functions. (**E**) Heatmap showing associations between differential immune cell functions and prognostic genes. *P < 0.05; **P < 0.01; ***P < 0.001. (**F**) Scatter plots illustrating the correlation between HS3ST4 and macrophages (left), and NRXN1 and memory B cells (right). Statistical significance is denoted as follows: ns, not significant. Abbreviation: GSEA: Gene Set Enrichment Analysis.

GSEA revealed significant enrichment of pathways related to ABC transporters, fatty acid metabolism, glycolytic glucose production, pyruvate metabolism, signal recognition particle (SRP)-dependent cotranslational protein targeting, ABC family protein-mediated transport, protein glycosylation disorders, mitochondrial fatty acid β-oxidation, and ubiquitin-mediated enzymatic roles of E1 and E2 enzymes (Fig. 4A). These results suggest that these pathways play a pivotal role in OS pathogenesis and progression. Furthermore, immune infiltration analysis (Fig. 4B) demonstrated significant differences in 11 immune cell populations and 3 immune functions between the two risk groups, all of which exhibited higher expression in the low-risk group. Notably, Mast cells, Macrophages, MDSCs, CD56bright Natural Killer (NK) cells, NK cells, Immature Dendritic Cells, and Central Memory CD4^+^ T cells were significantly elevated in the low-risk group (Fig. 4C). Interestingly, a strong positive correlation was observed between these differentially infiltrated immune cells and immune functions (Fig. 4D). Further correlation analysis between prognostic genes and immune cell infiltration revealed that NRXN1 and OR2W3 exhibited significant negative correlations with Central Memory CD4^+^ T cells, Central Memory CD8^+ T cells, Memory B cells, and Natural Killer T cells. Conversely, HS3ST4 and KLK2 showed significant positive correlations with Type 17 T Helper Cells, Neutrophils, and Immature Dendritic Cells (Fig. 4E). Notably, HS3ST4 displayed the strongest positive correlation with Macrophages (cor = 0.3551, *P* = 0.0009), while NRXN1 exhibited the strongest negative correlation with Memory B cells (cor = -0.3696, *P* = 0.0005) (Fig. 4F).

### Correlation of prognostic genes with immune checkpoint and TIDE

**Fig. 5:**
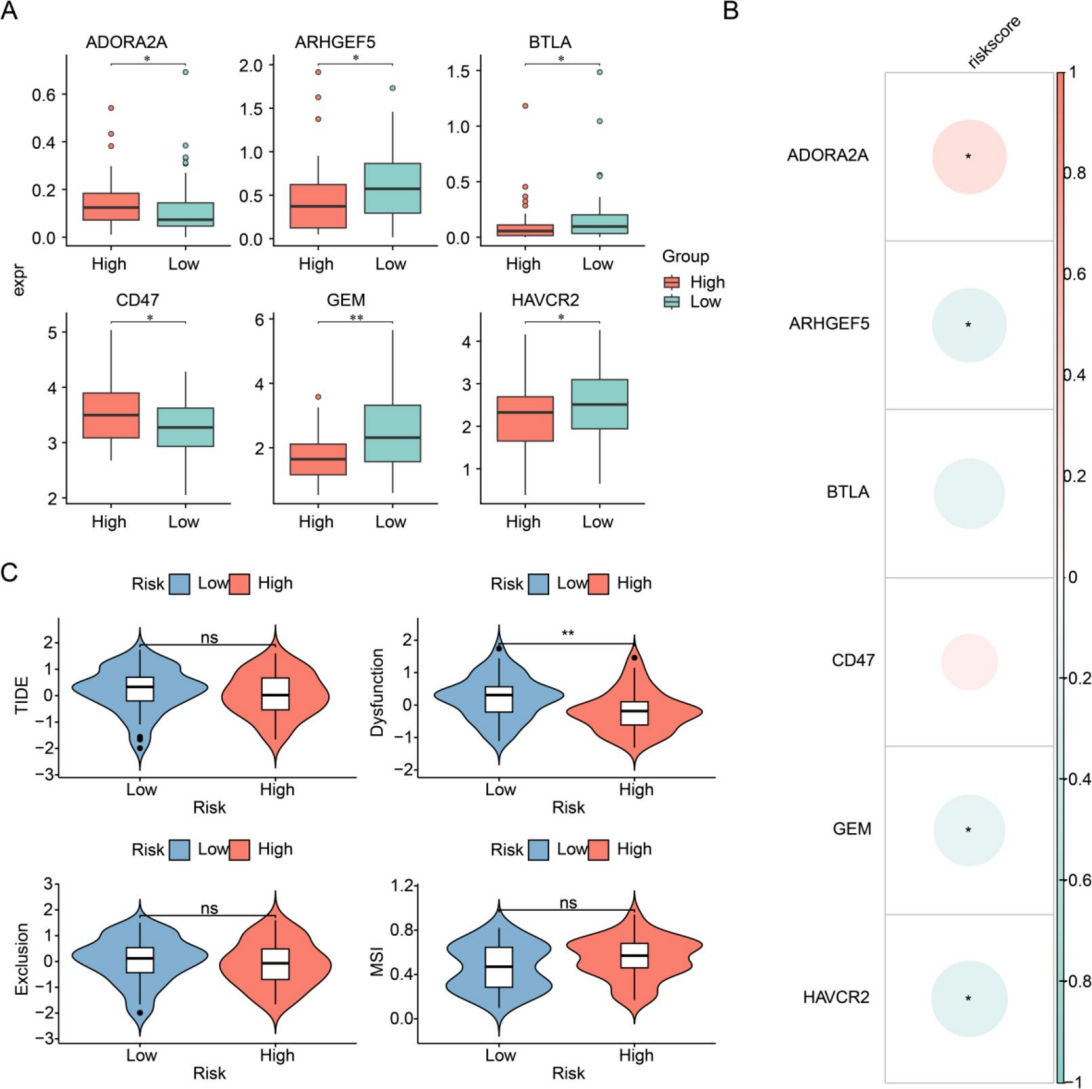
Immune checkpoint expression and tumor-immune function across risk groups. (**A**) Differential expression of immune checkpoints between the two risk groups. *P < 0.05; **P < 0.01. (**B**) Lollipop plot illustrating correlations between immune checkpoint expression and risk scores. *P < 0.05. (**C**) Comparison of TIDE, Dysfunction, Exclusion, and MSI scores between the two groups. Statistical significance: ns, not significant; **P < 0.01.

The Wilcoxon test identified significant differences in HAVCR2, ADORA2A, ARHGEF5, BTLA, CD47, and GEM expression between the two risk groups, and except for ADORA2A, the remaining five immune checkpoints were all highly expressed in the low-risk group (Fig. 5A). The lollipop plot further revealed that ADORA2A was significantly positively correlated with the risk score, while three immune checkpoints (GEM, HAVCR2, ARHGEF5) had a strong negative correlation between these immune checkpoints and the risk score (Fig. 5B). Additionally, Dysfunction differed markedly between risk groups, displaying lower levels in the high-risk group. This suggests reduced T cell dysfunction and partial preservation of immune function within the tumor microenvironment in high-risk patients (Fig. 5C).

### Study on the regulatory mechanism of prognostic genes on OS

**Fig. 6:**
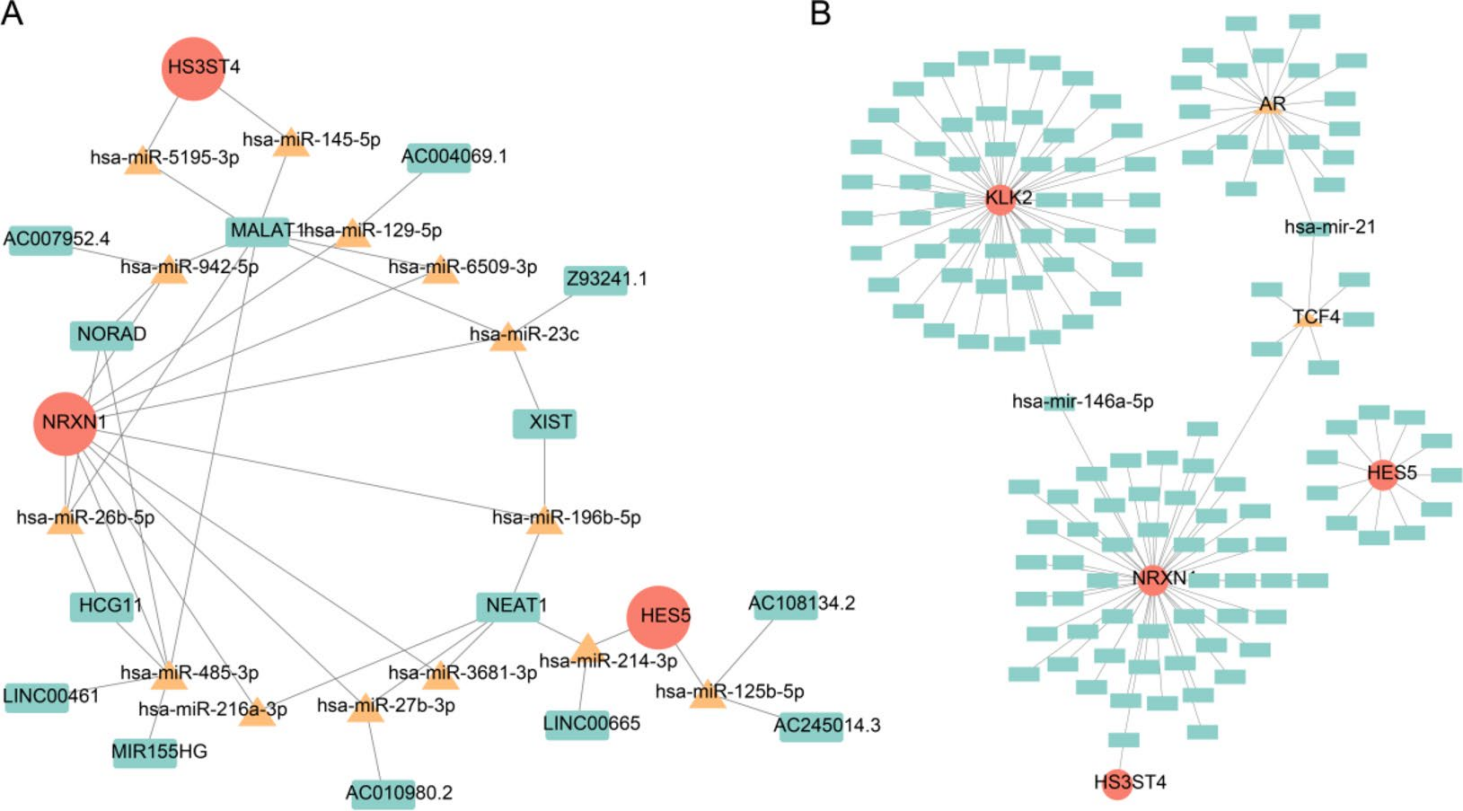
ceRNA-mediated regulatory mechanisms of prognostic genes. (**A**) mRNA-miRNA-lncRNA regulatory network, where red nodes represent prognostic genes, yellow triangles denote miRNAs targeting prognostic genes, and green squares indicate lncRNAs interacting with miRNAs. (**B**) TF-mRNA-miRNA regulatory network, with red nodes representing prognostic genes, yellow triangles indicating TFs, and green squares denoting miRNAs. Abbreviations: MDGs: mitochondrial autophagy-related differential genes; OS: osteosarcoma; TF: transcription factor.

To explore the regulatory mechanisms underlying prognostic genes in OS, a ceRNA network was constructed based on predicted miRNA and lncRNA interactions. NRXN1 was associated with 10 miRNAs and 11 lncRNAs, while HES5 correlated with hsa-miR-125b-5p and AC245014.3. Additionally, NRXN1 exhibited regulatory interactions with hsa-miR-26b-5p and NORAD, whereas HS3ST4 was linked to hsa-miR-145-5p and MALAT1 (Fig. 6A). Furthermore, 134 miRNAs and two TFs—TCF4 and AR—were predicted to regulate HES5, NRXN1, HS3ST4, and KLK2. Notably, hsa-miR-146a-5p simultaneously targeted KLK2 and NRXN1, while TCF4 modulated NRXN1 and hsa-miR-21 (Fig. 6B).

### The expression of prognostic genes was higher in the normal group

**Fig. 7:**
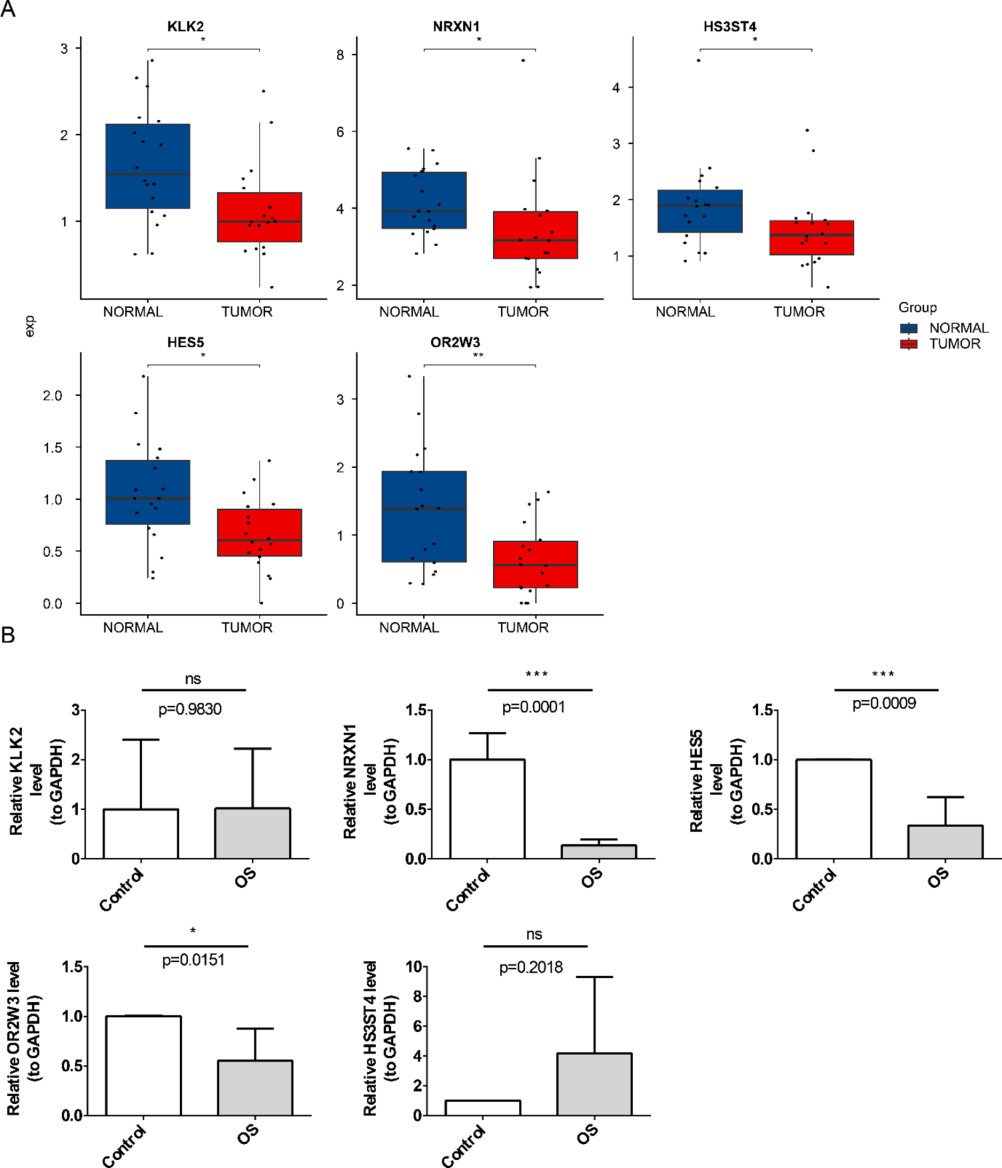
Prognostic gene expression in tumor and normal tissues. (**A**) Box plot illustrating prognostic gene expression in GSE99671. (**B**) RT-qPCR validation of prognostic gene expression. Statistical significance: ns, not significant; *P < 0.05; ***P < 0.001.

Analysis of GSE99671 revealed significantly higher expression levels of KLK2, NRXN1, HES5, OR2W3, and HS3ST4 in normal samples compared to tumor samples (Fig. 7A). Consistently, RT-qPCR validated the elevated expression of HES5, NRXN1, and OR2W3 in normal tissues, aligning with dataset findings (Fig. 7B).

## Discussion

Mitochondrial autophagy, first proposed in 2005, is a vital mechanism for eliminating damaged mitochondria in eukaryotic cells, thereby preserving essential cellular functions such as differentiation, homeostasis, and neuroprotection^25^. This process is triggered by hypoxia, nutrient deprivation, cellular senescence, and other stimuli, effectively limiting excessive ROS production to maintain mitochondrial network integrity and intracellular stability^8^. Mitochondrial autophagy is intricately linked to various human cancers, exhibiting differential expression in colorectal, breast, and lung cancers compared to normal tissues^10^. Its role in tumorigenesis and progression is paradoxical—it exerts tumor-suppressive effects by eliminating dysfunctional mitochondria and reducing ROS levels, thereby curbing tumor-initiating capacity^26^. Conversely, during tumor progression, mitochondrial autophagy supports tumor survival and facilitates malignant development^10^. Notably, inhibiting mitochondrial autophagy has been implicated in sensitizing tumor cells to chemotherapy, suggesting its potential as a therapeutic target^9,27^. Despite its significance, the role of mitochondrial autophagy in prognosis, immune response, and regulatory mechanisms in OS remains unclear. This study identified five MDGs—KLK2, NRXN1, HES5, OR2W3, and HS3ST4 and constructed a risk model capable of predicting OS survival outcomes. The high-risk group exhibited a poorer prognosis, underscoring the model’s clinical relevance. Notably, all five MDGs were significantly downregulated in tumor samples from GSE99671. RT-qPCR validation further confirmed the overexpression of NRXN1, HES5, and OR2W3 in adjacent non-tumor tissues, while KLK2 and HS3ST4 showed no significant difference. These findings demonstrate strong concordance between prognostic gene expression patterns and the risk model, reinforcing its reliability.

Kallikrein-related peptidase 2 (KLK2) is a serine protease belonging to the glandular kallikrein protein family (KLK family), with predominant expression in the prostate. It is widely recognized as a prognostic biomarker in prostate cancer^28^. Bonk et al. reported that reduced or absent KLK2 expression correlates with tumor progression and serves as an independent adverse prognostic factor in ERG-negative prostate cancers^29^. The NRXN1 gene encodes neurexin 1, a protein enriched in brain regions involved in cognition. Mutations in NRXN1 are associated with schizophrenia, autism spectrum disorder, and other neurodevelopmental abnormalities^30^. Recent studies have linked NRXN1 overexpression to tumor progression and poor survival in multiple malignancies, including prostate cancer, breast cancer, and Ewing sarcoma^31,32^. Sun et al. demonstrated that NRXN1 expression is higher in normal tissues than in colorectal cancer samples in TCGA, where it is regulated by DNA methylation, identifying it as a potential biomarker for colorectal cancer^33^. This finding aligns with the present study, which suggests a potential role for NRXN1 in OS, offering a new avenue for further molecular investigations. HES5, a member of the basic helix-loop-helix (bHLH) superfamily, acts as a downstream effector of the Notch signaling pathway and is primarily expressed in epithelial tissues, regulating neuronal differentiation^34^. Increasing evidence suggests that HES5 is involved in tumor progression across various cancers, including non-small cell lung cancer (NSCLC), breast cancer, neuroblastoma, and hepatocellular carcinoma^34,35^. The Notch pathway has also been implicated in OS carcinogenesis. Ji et al. demonstrated that doxorubicin induces apoptosis and inhibits OS cell proliferation by activating the Notch pathway, as evidenced by the upregulation of NOTCH1, HEY1, HES1, and HES5^36^. However, the precise role of HES5 in enhancing chemotherapy sensitivity in OS remains to be elucidated. Odorant receptors (ORs), a subfamily of G protein-coupled receptors (GPCRs), have recently been identified in various human tissues and cancers, suggesting potential roles beyond olfaction^37^. OR2W3, a member of the ORs subfamily, exhibits higher expression in human paracancerous thyroid tissues than in carcinoma tissues, consistent with RT-qPCR results. Activation of OR2W3 enhances cell invasion in the follicular thyroid cancer (FTC133) cell line without affecting migration^38^. Masjedi et al. reported significant upregulation of OR2W3 in invasive breast cancer, correlating with poor survival, suggesting its potential as a biomarker for breast cancer progression^39^. HS3ST4, a member of the heparan sulfate (HS)-modifying enzyme family, has been implicated in cancer progression and immune evasion^40^. Hosseinpour et al. identified hsa-miR-592 and its target gene HS3ST4 as potential diagnostic and therapeutic markers for early-stage breast cancer^41^. Elevated telomere repeat binding factor 2 (TRF2) expression has been widely observed in human cancers. Its upregulation promotes HS3ST4 expression, accelerating tumor growth in vivo and potentially contributing to immune evasion by inhibiting NK cell activation^42^.

To elucidate the potential mechanisms underlying the prognostic model, GSEA was performed between the two risk groups. The analysis identified biological processes and pathways potentially involved in OS progression *via* MDGs, including fatty acid metabolism and mitochondrial fatty acid β-oxidation. Fatty acid metabolism supplies essential structural phospholipids for cancer cell membrane integration and has been implicated in the progression of breast cancer, liver cancer, bladder cancer, OS, and other cancers^43^. Mitochondrial fatty acid β-oxidation (FAO), a critical multi-step process for metabolizing fat and sugar into ATP, has been linked to cancer cell proliferation, survival, drug resistance, and metastasis. Additionally, FAO contributes to immune suppression and the tumor-promoting microenvironment^44^.

Although the processes modulating tumor cell metabolism and progression to MARGs have received much attention in recent years, the potential regulatory role of MARGs in tumor immunity remains largely unexplored and poorly understood. The studies have reported that mitochondrial autophagy plays a crucial role in modulating anticancer immune responses^45–47^. Similarly, we found that many immune-related cells and functions were remarkably different between the two risk groups. It is reasonable to believe that MARGs are associated with tumor immunity. Immunosuppression is frequently observed in the TME, primarily attributed to the lack of tumor-specific antigens and the activation of immune-suppressive signaling pathways, such as PD-1/PD-L1 and cytotoxic T-lymphocyte-associated antigen-4 (CTLA-4)^48^. Most clinical trials involving single PD-1/PD-L1 or PD-1/PD-L1 combined with CTLA-4 antibodies have yielded unsatisfactory responses in patients with OS^49–51^. This may be attributed to the inherent heterogeneity within the tumor immune microenvironment (TIME) of OS. This study analyzed immune-related differences between high- and low-risk groups through ssGSEA, using TARGET-OS datasets, and found that patients in the low-risk group exhibited greater immune cell infiltration and a higher immune function score. Additionally, higher expression levels of HAVCR2 and PDCD1LG2 were observed in the low-risk cohort, suggesting that these patients may benefit more from immunotherapy. OS is classified as an immune-suppressed tumor type, with tumor-associated macrophages (TAMs) comprising over 30% of the immune cell infiltrate, while T cell presence remains limited. TAMs play pivotal roles in OS progression, including tumor growth, angiogenesis, metastasis, and immune evasion^52^. Our findings revealed a strong positive correlation between HS3ST4 expression and TAM presence, indicating that patients with OS in the low-risk group may experience improved immunotherapeutic outcomes if strategies targeting HS3ST4 to modulate TAM activity within the TME are implemented. Moreover, the high-risk group demonstrated significantly higher TIDE scores, suggesting that T cells are the key immune cells likely to respond to PD-1/PD-L1 or CTLA-4-based therapies in this cohort. These insights may inform personalized immunotherapy strategies for patients with OS.

Extensive research has established the critical roles of lncRNAs and miRNAs in OS progression. To elucidate the regulatory mechanisms of prognostic genes in OS, a ceRNA network was constructed, incorporating 16 miRNAs, 14 lncRNAs, and 5 highly correlated prognostic genes. HES5 was linked to hsa-miR-125b-5p and AC245014.3, NRXN1 to hsa-miR-26b-5p and non-coding RNA activated by DNA damage (NORAD), and HS3ST4 to hsa-miR-145-5p and MALAT1. Notably, miR-26b-5p is downregulated in OS, suppressing tumor proliferation, migration, and invasion, while also inhibiting OS metastasis by targeting CTGF and Smad1^53^. Conversely, NORAD is upregulated in OS tissues, repressing miR-155-5p and promoting tumor progression. The regulatory role of NRXN1 in OS appears intricate and warrants further investigation. Additionally, a TF-mRNA-miRNA network was established to explore TFs and miRNAs interacting with prognostic genes. Zhou et al. reported that miR-146a-5p is highly expressed in OS and positively correlates with tumor size and recurrence, facilitating OS progression *via* the ZNRF3/GSK-3β/β-catenin signaling pathway^54^. Likewise, TCF4 expression is elevated in doxorubicin (DXR)-resistant OS cells and tissues, enhancing tumor progression by inhibiting the Wnt/β-catenin pathway^55^. This study identified hsa-miR-146a-5p as a regulator of KLK2 and NRXN1, while TCF4 was found to modulate NRXN1 and hsa-miR-21. These findings suggest that the hsa-miR-146a-5p-KLK2/NRXN1 and TCF4-NRXN1/hsa-miR-21 regulatory axes may play pivotal roles in OS development, necessitating further validation.

This study has several limitations. Firstly, OS is a multi-gene mutation disease, and the prognostic model we constructed based on MDGs inherently has bias. Secondly, this study constructed the model using transcriptome data and clinical information from public databases. Although the results showed a correlation between mitochondrial autophagy and OS, there is a lack of direct experimental evidence. Based on this, in the future, we plan to conduct in vitro experiments in OS cell lines, using gene editing techniques to regulate the expression of genes related to mitochondrial autophagy, and deeply observe changes in cell proliferation, apoptosis, migration, and invasion capabilities, as well as their impact on immune cell function. Additionally, we will construct an animal model to further validate the role of mitochondrial autophagy in tumor growth, metastasis, and the immune microenvironment in vivo. Through these in vivo and in vitro experiments, we can more comprehensively and deeply investigate the mechanism of mitochondrial autophagy in OS, thereby providing more solid experimental evidence for our research results.

## Conclusion

This study identified MDGs associated with OS prognosis using bioinformatics and screened five survival-related genes. Furthermore, the risk score exhibited associations with immune response and regulatory mechanisms, suggesting potential as prognostic biomarkers and therapeutic targets in OS.

## Data Availability

The sample data of OS were obtained from the Gene Expression Omnibus (GEO) database (https://www.ncbi.nlm.nih.gov/geo/) with accession number GSE99671 and GSE21257 The TARGET-OS related genes and clinical information of samples were obtained from the UCSC Xena (http://xena.ucsc.edu/) The mitochondrial autophagy related genes were obtained from the Reactome database (https://reactome.org/).

## Abbreviations

OS: Osteosarcoma
GEO: Gene expression omnibus
TCGA: The cancer genome atlas
DEGs: Differentially expressed genes
GSEA: Gene set enrichment analysis
MARGs: Mitochondrial autophagy related genes
ssGSEA: Single-sample gene set enrichment analysis
MDGs: Mitochondrial autophagy-related differential genes
UCSC: University of California, Santa Cruz
GSVA: Gene set variation analysis
GO: Gene ontology
KEGG: Kyoto encyclopedia of genes and genomes
HR: Hazard ratios
LASSO: Least absolute shrinkage and selection operator
K-M: Kaplan-meier
